# Stimulators and Activators of Soluble Guanylate Cyclase: Review and Potential Therapeutic Indications

**DOI:** 10.1155/2012/290805

**Published:** 2012-02-28

**Authors:** Bobby Nossaman, Edward Pankey, Philip Kadowitz

**Affiliations:** ^1^Critical Care Medicine Section, Department of Anesthesiology, Ochsner Medical Center, 1514 Jefferson Highway, New Orleans, LA 70121, USA; ^2^Department of Pharmacology, SL83, Tulane University School of Medicine, 1430 Tulane Avenue, New Orleans, LA 70112-2699, USA

## Abstract

The heme-protein soluble guanylyl cyclase (sGC) is the intracellular receptor for nitric oxide (NO). sGC is a heterodimeric enzyme with **α** and **β** subunits and contains a heme moiety essential for binding of NO and activation of the enzyme. Stimulation of sGC mediates physiologic responses including smooth muscle relaxation, inhibition of inflammation, and thrombosis. In pathophysiologic states, NO formation and bioavailability can be impaired by oxidative stress and that tolerance to NO donors develops with continuous use. Two classes of compounds have been developed that can directly activate sGC and increase cGMP formation in pathophysiologic conditions when NO formation and bioavailability are impaired or when NO tolerance has developed. In this report, we review current information on the pharmacology of heme-dependent stimulators and heme-independent activators of sGC in animal and in early clinical studies and the potential role these compounds may have in the management of cardiovascular disease.

## 1. Introduction

**Figure 1:**
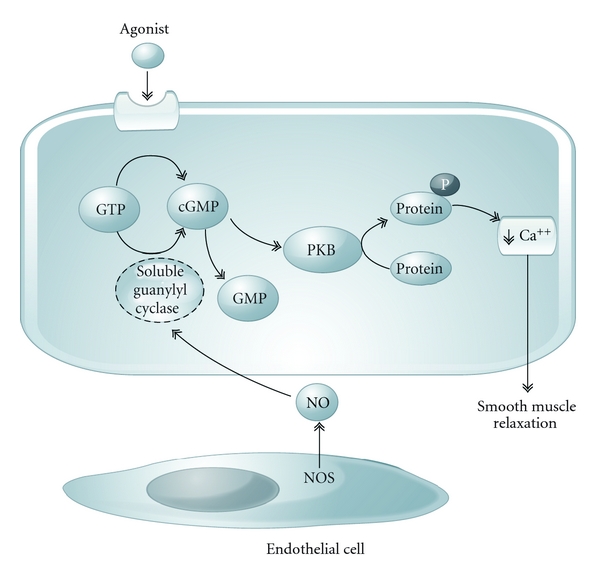
Simplified role of NO (nitric oxide) stimulating soluble guanylyl cyclase smooth muscle relaxation. PKB (protein kinase B), NOS (nitric oxide synthase).

Guanylyl cyclase (GC) is an enzyme that catalyzes the formation of guanosine 3′,5′-monophosphate (cGMP) from guanosine triphosphate (GTP) and is found in tissues throughout the animal kingdom [1, 2]. Soluble GC (sGC) is the receptor for nitric oxide (NO) in vascular smooth muscle [3, 4]. In the cardiovascular system, NO is endogenously generated by endothelial NO synthase (eNOS) from L-arginine and activates sGC in adjacent vascular smooth muscle cells to increase cGMP levels and induce relaxation (Figure 1). NO plays a major role in the regulation of vascular tone and blood pressure [5, 6]. When released from the endothelium in response to physiologic stimuli such as shear stress, NO binds to the normally reduced heme moiety of sGC and increases the formation of cGMP from GTP leading to a decrease in intracellular calcium and vasodilation [7–10]. Moreover, the NO-sGC-cGMP pathway is essential for the control of a number of physiologic processes, including neuronal transmission, host defense, cell growth and proliferation, and vascular and platelet homeostasis [11–18].

## 2. History

**Figure 2:**
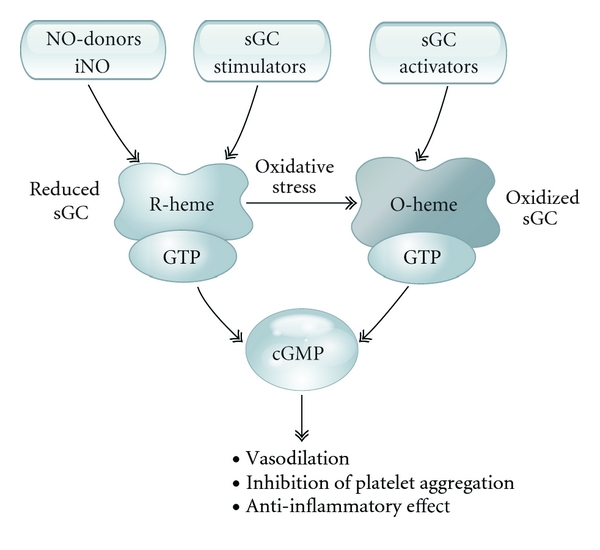
Role of NO (nitric oxide), inhaled NO, and sGC (soluble guanylyl cyclase) stimulators in stimulating the reduced heme of sGC and the role of sGC activators in stimulated oxidized sGC to stimulate cGMP leading to vasodilation, inhibition of platelet aggregation and an anti-inflammatory effect in the vascular bed.

Initial investigations into the role of NO were conducted showing that nitrogen-containing compounds such as sodium azide (NaN_3_), sodium nitrite (NaNO_2_), hydroxylamine (NH_2_OH), nitroglycerin (C_3_H_5_N_3_O_9_), and sodium nitroprusside (Na_2_[Fe(CN)_5_NO]) were able to activate GC [3, 19–23] (Figure 2). When tissues were homogenized and separated by centrifugation, GC activity was detected in particulate and soluble fractions [19, 23–31]. As NO was shown to rapidly activate GC [3], it was hypothesized that GC activation may be due to the effect of NO or another substance that activated the enzyme [19]. Moreover, these nitrogen-containing compounds were able to activate sGC, causing an increase in cGMP, and vascular relaxation [13, 21, 32, 33].

## 3. sGC in Health and Cardiopulmonary Disease

Reduced bioavailability and/or responsiveness to endogenous NO have been implicated in the pathogenesis of many disease processes [34–36]. NO was originally described as an endothelial-derived relaxing factor and is a vasodilator in the pulmonary and systemic circulations [37–40]. The significance of NO in the regulation of vasomotor tone has been demonstrated in experimental animals and in human subjects by the use of NOS inhibitors [41–48]. Given the importance of the NO-sGC-cGMP pathway in cardiopulmonary diseases, there have been enormous efforts to improve NO therapy [36, 49–53]. 

### 3.1. Nitrates

Although glyceryl trinitrate (GTN) and amyl nitrite have been used in the treatment of angina pectoris and heart failure for over 140 years [54, 55], the most commonly used agents at the present include isosorbide dinitrate, isosorbide-5-mononitrate, and GTN which are effective in reducing ventricular preload by increasing peripheral venous capacitance [56–59]. It is generally believed that the therapeutic effect of these drugs involves the release of NO from nitrite anion, the activation of sGC, and relaxation of capacitance blood vessels [54, 60–63]. These drugs can also decrease pulmonary and systemic vascular resistances but require higher doses than needed for increasing venous capacitance [64–69]. These agents can reduce ventricular filling pressure, wall stress, and myocardial oxygen consumption [70] and may also improve systolic and diastolic ventricular function by improving coronary flow in patients with ischemic cardiomyopathy. However, there is as yet no convincing evidence that organic nitrates improve mortality in patients with acute myocardial infarction [71, 72]. The limitations of this class of agents are well known and include adverse hemodynamic effects, including tolerance, lack of selectivity, and limited bioavailability [63].

Studies in the literature provide evidence that vasorelaxant responses to GTN are mediated by the formation of NO or aclosely related S-nitroso molecule [13, 33, 73, 74]. However, the mechanism of this vasorelaxant response to GTN is uncertain. Although studies in the literature indicate that NO contributes to the activation of sGC and vascular smooth muscle relaxation [75, 76], other studies suggest that vasorelaxant responses to GTN may be independent of NO release and cGMP formation [77].

Studies have shown that the bioactivation of GTN requires the presence of thiols or sulfhydryl-containing compounds and that NO or NO-like compounds are believed to be the biologically active species [3, 74, 78, 79]. Interactions with GTN and sulfhydryl-containing molecules are necessary for vascular smooth muscle relaxation in that repeated administration of GTN produces sulfhydryl depletion and the development of tolerance [79–81]. Subsequent studies have demonstrated the release of NO following the decomposition of an intermediate S-nitrosothiol [74]. Additional studies suggest that an enzymatic mechanism may be responsible for the bioactivation of GTN. However, these enzymatic systems could not catalyze the selective formation of 1,2-glyceryl dinitrate and nitrite from GTN, and moreover, no association between the development of enzymatic tolerance and tolerance to GTN was observed [82–88]. Moreover, the discovery (1) that mitochondrial aldehyde dehydrogenase (mtALDH) generates 1,2-glyceryl dinitrate and nitrite from GTN [89], (2) that this reaction requires a reducing thiol cofactor [60, 90, 91], and (3) that the activity of this enzyme is reduced in GTN tolerance [53, 61] suggests that this pathway is responsible for GTN bioactivation in vascular smooth muscle [53, 77, 89]. However, one difficulty with these studies has been an inability to detect NO as a byproduct of GTN metabolism [92]. Moreover, the generation of NO was observed when the concentrations of GTN exceeded therapeutic values [93–98]. A potential solution has been proposed that once GTN is bioactivated within the mitochondria, nitrite or an additional action of mtALDH generates the vasodilatory NO bioactivity [53]. One suggested mechanism for this vasodilatory activity is that S-nitrosoglutathione is formed by the reaction of reduced glutathione and nitrite [99, 100]. This molecule subsequently undergoes biotransformation to S-nitrosocysteine [89, 101] that can release NO [102]. However, excessive amounts of GTN or S-nitrosothiols can dysregulate protein S-nitrosylation and contribute to cellular dysfunction and disease [103]. Chronic GTN administration has been shown to result in acetylcholine-induced coronary vasoconstriction rather than relaxation [104, 105] and induce endothelial dysfunction [106]. An early event in the development of atherosclerosis is the impairment of endothelial function or endothelial dysfunction that develops before structural changes and intimal hyperplasia or lipid deposition occur [107]. Moreover, reduced oxidation of NO occurs through altered endothelial NOS formation and activity [107], which can be evidenced by abnormal vascular responses to an acetylcholine challenge [108, 109]. Therefore, NO deficiency is linked to cardiopulmonary disease processes and provides justification for the use of effective NO replacement therapy.

## 4. Activation of sGC by NO-Like Compounds

The activation of sGC enhances the conversion of GTP to cGMP that mediates physiologic responses including smooth muscle relaxation and inhibition of platelet aggregation [13–18]. However, little is known about sGC regulation by substances other than NO-donors. The recent discovery of a benzylindazole derivative, YC-1, that was shown to inhibit platelet aggregation and increase by sixfold intracellular concentrations of cGMP, opened up new areas of research in sGC regulation [110, 111]. Subsequent studies found that activation of sGC by YC-1 was NO-independent and was independent of biotransformation [112, 113]. In contrast, organic nitrates appear to require biotransformation with intermediates (nitrites, nitrosothiols, and organic thionitrates) liberating NO [91, 114]. These findings with YC-1 as well as other stimulators of sGC suggest that non-NO compounds may also activate or modulate sGC activity [115–118]. Current NO-donor drugs induce tolerance [34–36, 79–81]. However by increasing the responsiveness of sGC to endogenous NO, YC-1 or YC-1-like compounds may represent a novel class of drugs that cansensitize the sGC enzyme to respond to NO. In disease states with dysfunctional sGC, it may be possible to increase the effect of endogenously produced NO or improve the effects of NO-donor drugs. NO-independent stimulators for sGC have been developed for use in pathophysiologic conditions when NO formation and bioavailability are impaired or when NO tolerance has developed [34–36].

### 4.1. sGC Stimulators

#### 4.1.1. Preclinical Studies

Compounds have been developed that can directly stimulate sGC and increase cGMP formation in pathophysiologic conditions when NO formation and bioavailability are impaired or when NO tolerance has developed [34–36] (Figure 2). The pyrazolopyridine compound, BAY 41-8543, is an NO-independent stimulator of sGC that has been shown to reduce systemic and pulmonary arterial pressure, and relax isolated vessels from a variety of organ systems [35, 119, 120]. BAY 41-2272 is closely related to BAY 41-8543, and this sGC stimulator has been shown to have significant pulmonary vasodilator activity in a variety of species [119, 121–125]. BAY 41-2272 has been shown to reduce right ventricular hypertrophy and pulmonary vascular remodeling in a chronic hypoxia-induced model of pulmonary hypertension [126]. It has been reported that when either BAY 41-2272 or BAY 41-8543 was administered by inhalation to awake lambs, the pyrazolopyridine compounds had selective pulmonary vasodilator activity and BAY 41-8543 could enhance the magnitude and prolong the duration of the vasodilator response to inhaled NO [122, 125].

In an intact chest rat model, administration of BAY 41-8543 under control or baseline tone conditions produced small decreases in pulmonary arterial pressure, larger dose-dependent decreases in systemic arterial pressure, and increases in cardiac output [127]. However, under elevated tone conditions induced with the thromboxane receptor agonist, U46619, BAY 41-8543 produced larger dose-dependent decreases in pulmonary arterial pressure when tone in the pulmonary vascular bed was increased [127]. Analyses of the percent decreases in pulmonary and systemic arterial pressures in response to BAY 41-8543 under elevated tone conditions induced with U46619-infused animals were not different, suggesting that the sGC stimulator had similar vasodilator activity in the pulmonary and systemic vascular beds in the intact chest rat [127].

The effect of NOS inhibition with L-NAME on vasodilator responses to BAY 41-8543 was investigated in the intact chest rat model, and following administration of the NOS inhibitor, decreases in pulmonary and systemic arterial pressures in response to BAY 41-8543 were reduced when compared to responses in U46619-infused animals. Comparisons of responses to BAY 41-8543 at the same level of pulmonary arterial pressure indicate that decreases in pulmonary arterial pressure in response to the sGC stimulator are reduced by more than 50% in L-NAME-treated animals [127]. These results are consistent with the concept that responses to the sGC stimulator are NO-independent; however, in the absence of endogenous NO formation, vasodilator responses to the sGC stimulator were markedly attenuated. It has been reported that stimulators of sGC have a dual role of action in that they directly stimulate the native form of the enzyme and render it more sensitive to endogenously produced NO or augment the action of exogenously administered NO [125, 128]. The results in the intact chest rat model are consistent with these findings [125, 128] that show that vasodilator responses to BAY 41-8543 are attenuated when endogenous NO formation is inhibited.

The role of BAY 41-8543 synergy with exogenous NO was examined in the intact chest model with the NO donor, sodium nitroprusside (SNP). Although separate administration of BAY 41-8543 and SNP produced significant decreases in pulmonary and systemic arterial pressures, coadministration of a small dose of the NO donor along with BAY 41-8543 produced decreases in pulmonary and systemic arterial pressures that were significantly greater than the sum of responses to either agent when administered alone [127]. These results suggest that BAY 41-8543 synergizes with NO in mediating vasodilator responses to the sGC stimulator in the pulmonary and systemic vascular beds in the intact rat.

BAY 41-8543 and BAY 41-2272 were synthesized based upon analysis of the structure of YC-1 [36, 110, 129]. These pyrazolopyridine stimulators activate sGC in a manner independent of NO [35, 36, 121]. These compounds activate purified sGC and strongly synergize with NO, reflecting stabilization of the nitrosyl-heme complex of the enzyme [130]. Both BAY 41-8543 and BAY 41-2272 relax vascular smooth muscle and have vasodilator activity in the pulmonary and systemic vascular beds [34, 119, 121, 122, 128, 131].

Although BAY 41-8543 had beneficial effects in experimental models of pulmonary hypertension, this agent does not have favorable pharmacokinetic properties and cannot be used in clinical trials [132]. In contrast, BAY 63-2521 (Riociguat; Bayer Healthcare AG, Wuppertal, Germany), a heme-dependent sGC stimulator closely related to BAY 41-8543, has a better pharmacokinetic profile and has been used in clinical studies [132]. In respect to interesting similarities and differences between the actions of BAY 41-8543 and other NO-independent stimulators of sGC, it has been reported that BAY 41-2272, which is chemically similar to BAY 41-8543, produced greater decreases in pulmonary than systemic arterial pressure and that pulmonary vasodilator responses were not attenuated by L-NAME [125]. In the present study, BAY 41-8543 produced similar decreases in pulmonary and systemic arterial pressures in U46619-infused animals and decreases in both pulmonary and systemic arterial pressures attenuated by L-NAME treatment. In an ovine fetal model of pulmonary hypertension, chronic infusion of BAY 41-2272 produced potent sustained decreases in pulmonary arterial pressure that were not attenuated by a NOS inhibitor, L-NA, and when infused at higher rates, systemic arterial pressure was decreased [124]. The main differences in response to stimulators of sGC in the awake sheep, the ovine fetal circulation, and intact chest rat are the relative differences in vasodilator activity in the pulmonary and systemic vascular beds and the role of endogenously produced NO in modulating these responses [124, 125]. The reason for the differences in results in the different experimental models may involve differences in species, experimental design and preparation, the BAY compound studied, or more importantly, the mechanisms involved in the stimulation of sGC. In the present study, BAY 41-8543 had similar vasodilator activity in the preconstricted pulmonary vascular bed and the systemic vascular bed and vasodilator responses in both beds were attenuated when NOS was inhibited with L-NAME. These data suggest that the role of endogenous NO in the activation of sGC is similar in both circulations in the intact chest rat model and may differ from SGC activation mechanisms in the pulmonary and systemic vascular beds in the awake sheep and ovine fetal preparation [124, 125].

Although pulmonary vasodilator response to the sGC stimulator, BAY 41-2272, was not dependent on the formation of endogenous NO in the awake sheep model, the response strongly synergized with inhaled NO [125]. Therefore, the ability of the sGC stimulator to synergize with exogenous NO was, in some respects, similar in the awake sheep and intact rat models, although the synergism was much greater in the awake sheep when NO was administered by inhalation [125]. The present results are consistent with the concept that sGC stimulators can be given along with inhaled NO or an infused NO-donor to produce maximum pulmonary vasodilation [125, 128].

#### 4.1.2. Clinical Investigations

The first sGC simulator to undergo clinical study was BAY 41-8543 [34]. However, although systemic blood pressure decreased as expected following oral administration in healthy volunteers, other pharmacokinetic issues occurred that lead to the development of additional sGC compounds [34]. Subsequently BAY 63-2521 was developed, and when administered in 58 healthy male volunteers as a single oral dose (0.25–5 mg), no serious adverse events were observed [133]. Although both mean arterial and diastolic pressures were decreased, systolic pressure was not significantly affected. A dose-related increase in heart rate up to ~11 bpm was observed with the 5-mg dose; however, this dose was not well tolerated, due to an increased number of adverse events, including headache, nasal congestion, flushing, feeling hot, orthostatic hypotension, and palpitations. Increased levels in the vasoactive hormones, norepinephrine, and plasma renin, but not plasma aldosterone or angiotensin II, were observed [133]. Following these encouraging findings, a clinical study was performed to evaluate the short-term safety profile of BAY 63-2561 (Riociguat) to determine the tolerability and efficacy in patients with moderate to severe pulmonary hypertension (PH) due to pulmonary arterial hypertension, distal chronic thromboembolic PH, or PH with mild to moderate interstitial lung disease [134]. Safety and tolerability studies were performed in 19 subjects with single doses ≤2.5 mg. The administration of the sGC stimulator significantly improved pulmonary hemodynamic measurements and cardiac indices in patients with PH in a dose-dependent manner, to a greater extent than following administration of inhaled NO. Although riociguat had significant systemic blood pressure effects and demonstrated no selectivity for the pulmonary circulation, however, mean systolic blood pressure remained >110 mmHg [134]. The drug was well tolerated and found to be superior to inhaled NO in the response of the pulmonary circulation [134]. In a 12-week, phase II study, 75 patients with a diagnosis of PH (chronic thromboembolic pulmonary hypertension or pulmonary arterial hypertension) received oralriociguat in 0.5 mg increments in 2-week intervals from 1 mg to a maximum of 2.5 mg three times a day that was titrated to systemic systolic blood pressure (SBP) [135]. The dose of the sGC stimulator was increased if systemic blood pressure was greater than 100 mmHg, was maintained once SBP was stable in a range of 90–100 mmHg, and decreased if SBP was less than 90 mmHg or with symptoms such as syncope or dizziness. The primary endpoints studied were safety and tolerability of the sGC stimulator with changes in pharmacodynamics as the secondary endpoints. Riociguat was well tolerated, and that asymptomatic hypotension (SBP less than 90 mmHg) occurred in 11/75 (15%) patients, but blood pressure could be normalized with dose alteration in 2 patients and without dose alteration in 9 patients [135]. Pulmonary vascular resistance was significantly reduced. A significant improvement in the median 6-minute walking distance was observed in patients with diagnosis of chronic thromboembolic pulmonary hypertension (greater than 55 meters; *P* < 0.0001) and in patients with pulmonary arterial hypertension (PAH) (greater than 57 meters; *P* < 0.0001). Moreover, similar improvements were also observed in patients on chronic bosentan therapy [135]. The most frequent observed adverse events were dyspepsia, headache, and hypotension but were considered mild or moderate in 96% in these patients. Only in 4% of patients was the medication discontinued. In this 12-week study, the sGC stimulator demonstrated a favorable safety profile with significant improvements in pulmonary hemodynamics, and in exercise capacity, but with a high through mild to moderate incidence of patient symptoms [135].

### 4.2. sGC Activators

#### 4.2.1. Preclinical Studies

The oxidation of the heme iron on sGC decreases the responsiveness of the enzyme to NO and promotes vasoconstriction [136]. Recently, stimulatory compounds with a different mode of sGC activation have been developed and have been shown to target NO receptor proteins when the heme iron on sGC is in an oxidized (Fe^3+^ instead of Fe^2+^) state, or when the heme group is lost [36, 131, 137, 138] (Figure 2). These compounds activate the oxidized or heme-deficient sGC enzyme that is not responsive to NO [51]. Oxidation of sGC results in loss of activation of the enzyme [139–142]. Moreover, purified sGC also results in marked to complete loss of enzyme responsiveness to NO-donors [143, 144]. However, responsiveness was restored by the addition of hematin, hemoglobin, or a heat-inactivated catalase in the presence of a reducing agent [143, 144]. Organic nitrates have been used in the treatment of angina, but the development of tolerance limits their therapeutic value [53, 60, 145–147]. The development of compounds that overcome this limitation that are able to stimulate sGC independent of redox state as shown in tissue preparations and *in vivo* studies indicates that these NO-independent receptors exist and may become more abundant under pathological conditions associated with oxidative stress [148–150].

Activation of the NO-sGC-cGMP pathway can induce potent pulmonary and systemic vasodilatation [40, 151–155]. Two classes of novel drugs have been developed that can modulate sGC-cGMP signal transduction in an NO-independent manner. Although stimulators of sGC can enhance the sensitivity of reduced sGC to NO [127], activators of sGC can increase sGC enzyme activity even when the enzyme is oxidized and less, or not responsive, to NO [34, 51, 52, 156]. In the intact chest rat, intravenous administration of the sGC activator, BAY 60-2770, produced dose-related decreases in systemic arterial pressure, increases in cardiac output, and decreases in systemic vascular resistance, and the cardiovascular responses were slow in onset and long in duration [157]. These observations are comparable to the findings in the anesthetized dog following administration of another sGC activator, BAY 41-2272 [123]. These findings suggest that sGC activators can increase sGC enzyme activity in vascular beds from different species [123, 157].

Following a preclinical study in rats demonstrating that Cinaciquat was able to reduce oxidative stress, improve cardiac performance, and improve impaired cardiac relaxation in experimentally induced myocardial infarction in rats [158], the role of the sGC activator in ischemia-reperfusion injury was investigated in a canine model of cardioplegic arrest and extracorporeal circulation [159]. Preconditioning with the sGC activator improved left- and right-ventricular contractility and led to a higher coronary blood flow. Moreover, endothelium-dependent vasodilatation to acetylcholine was improved. These findings suggest that preconditioning with Cinaciguat could improve myocardial and endothelial function following cardiopulmonary bypass and could be a novel therapeutic option in the protection against ischemia-reperfusion injury in cardiac surgery [159].

Vasodilator responses to this novel class of drugs have been studied in experimental models under conditions of acute pulmonary hypertension (PH) induced with the stable endoperoxide analogue, U46619 [122, 128]. In the intact chest rat under elevated pulmonary arterial tone conditions induced with U46619, administration of BAY 60-2770 produced significant decreases in both pulmonary and systemic arterial pressure [157]. Intravenous infusion of the sGC stimulator, BAY 41-2272, or inhalation of the sGC activator, BAY 58-2667, was able to reduce mean pulmonary arterial pressure and pulmonary vascular resistance in awake lambs [122, 128]. However, in contrast to the modest systemic pressure changes observed following administration of the sGC activator, BAY 58-2667, in the awake lamb [128], significant decreases in systemic arterial pressure were observed following administration of the sGC activator, BAY 60-2770, in the intact chest rat model [157]. The differences in results could be due to the type of sGC activator administered, experimental conditions, or species studied.

#### 4.2.2. Clinical Studies

The development of heart failure due to a number of etiologies is a common final stage in cardiovascular disease that is associated with high morbidity and mortality [160]. Stimulation of sGC with conventional nitrosovasodilators has been used for more than a century, but the development of tolerance, due to the prosthetic heme group of sGC existing in an oxidized or heme-free state, limits their clinical effectiveness [161, 162]. BAY 58-2667 (Cinaciguat; Bayer Healthcare AG, Wuppertal, Germany) has been shown to preferentially activate sGC when the prostetic heme group is in an oxidized or heme-free state [131]. As a result, Cinaciguat induces cGMP generation and vasodilation preferentially in diseased vessels [35, 121, 149] and has the potential to induce vasodilation and increase cardiac output in patients with HF. In the first clinical study with this sGC activator, the safety, tolerability, pharmacokinetics, and pharmacodynamics were analyzed in 76 healthy volunteers [163]. An intravenous infusion of Cinaciguat in a range of 50 to 250 mcg/h was administered for up to 4 hours. During the infusion period, the sGC activator decreased diastolic blood pressure and increased heart rate without significantly reducing systolic blood pressure. No serious adverse events were observed. However, at the higher infusion rates (150–250 mcg/h), a decrease in mean arterial pressure and an increase in plasma cGMP levels were observed. The findings that Cinaciguat had potent cardiovascular effects by reducing both preload and after load suggested that further investigation should occur in patients with HF [163]. In a multicenter phase II study, patients with a diagnosis of acute decompensated heart failure received 6-hour intravenous infusions of Cinaciguat which produced significant reductions in pulmonary capillary wedge pressure, mean right atrial pressure, mean pulmonary artery pressure, pulmonary vascular resistance, and systemic vascular resistance, and cardiac output by 1.7 L/min while only increasing heart rates by 4 bpm [164]. Cinaciguat was well tolerated in these patients, with approximately one-fourth of the patients reporting adverse events of mild to moderate intensity, with hypotension as the most common adverse event [164]. These results clearly demonstrate the clinical efficacy of the sCG activator.

## 5. Clinical Correlations

As sGC stimulators have been shown to stimulate sGC independently of NO donors, this class of agonists could have an important role in the acute hospital setting when a sudden increase in pulmonary vascular tone can occur during sympathetic overstimulation following major surgery that would be refractory to conventional NO-donor therapy, such as in patients who develop ARDS and are refractory to inhaled NO (iNO). The application of sGC stimulators would then make iNO therapy more effective in the treatment of acute pulmonary hypertension and possibly decease the incidence of associated right ventricular heart failure.

In contrast to the benefits from sGC stimulators, the application of sGC activators may have an important role in the therapeutic management of chronic-diseased blood vessels with extended periods of efficacy such as seen in systemic hypertension, atherosclerosis, diabetes mellitus, angina, and heart failure. Moreover, the use of this class of drugs may reduce the development of oxidative stress from NO-donor therapy and may reduce the common side effects from NO-donor therapy such as headache that would improve patient compliance.

## 6. Conclusion

Research has shown that heme-dependent drugs are effective in the treatment of cardiopulmonary disorders, but that their effects are diminished under pathological conditions associated with increased oxidative stress and the development of tolerance. In the past decade, the development of heme-independent compounds has been shown to higher affinities for the oxidized form of sGC, and they are being developed for the treatment of acute decompensated heart failure and pulmonary hypertension. These two classes of drugs, sGC stimulators and sGC activators, have been studied in animal studies. These compounds are now undergoing preliminary clinical trials and may be available for clinical use within the near future.
